# Mesenchymal stromal cell therapies: immunomodulatory properties and clinical progress

**DOI:** 10.1186/s13287-020-01855-9

**Published:** 2020-08-08

**Authors:** Xiaomo Wu, Ju Jiang, Zhongkai Gu, Jinyan Zhang, Yang Chen, Xiaolong Liu

**Affiliations:** 1 Dermatology Institute of Fuzhou, Dermatology Hospital of Fuzhou, Xihong Road 243, Fuzhou, 350025 China; 2 Department of Biomedicine, University of Basel, Klingelbergstr 70, CH-4056 Basel, Switzerland; 3 The Institute of Biomedical Sciences, Fudan University, Mingdao Building, Dongan Road 131, Shanghai, 200032 China; 4 The United Innovation of Mengchao Hepatobiliary Technology Key Laboratory of Fujian Province, Mengchao Hepatobiliary Hospital of Fujian Medical University, Xihong Road 312, Fuzhou, 350025 China

**Keywords:** MSCs, Immunomodulatory activity, Paracrine effects, Cellular therapy

## Abstract

Mesenchymal stromal cells (MSCs) are a subset of heterogeneous non-hematopoietic fibroblast-like cells that can differentiate into cells of multiple lineages, such as chondrocytes, osteoblasts, adipocytes, myoblasts, and others. These multipotent MSCs can be found in nearly all tissues but mostly located in perivascular niches, playing a significant role in tissue repair and regeneration. Additionally, MSCs interact with immune cells both in innate and adaptive immune systems, modulating immune responses and enabling immunosuppression and tolerance induction. Understanding the biology of MSCs and their roles in clinical treatment is crucial for developing MSC-based cellular therapy for a variety of pathological conditions. Here, we review the progress in the study on the mechanisms underlying the immunomodulatory and regenerative effects of MSCs; update the medical translation of MSCs, focusing on the registration trials leading to regulatory approvals; and discuss how to improve therapeutic efficacy and safety of MSC applications for future.

## Introduction

Prior to being coined as mesenchymal stem cells by Caplan [1], mouse marrow-derived fibroblasts were exploited as feeder cells for long-term culture of hematopoietic stem cells, and Friedenstein et al. found, apart from niche-like properties, these cells are capable of generating bone/reticular tissue, cartilage, and fat [2–6]. Subsequently Pittenger et al. established that human bone marrow (BM) also contains a subpopulation of stromal cells exhibiting trilineage mesenchymal potential, differentiating into adipocytes, chondroblasts, and osteoblasts under defined condition in vitro [7]. Since then, these multipotent stromal cells have been isolated from a variety of tissues other than BM, including skeletal muscle, adipose tissue (AT), dental pulp, tendon, Wharton’s jelly, umbilical cords, amniotic fluid, and placentae, literately nearly all tissues but essentially from perivascular fraction [8]. Notably, the MSCs acronym has been collectively referred to as mesenchymal stem cells, multipotential stromal cells and mesenchymal stromal cells.

At present, identifying and characterizing MSCs are mostly via in vitro work based on the ability of adhering to plastic culture dishes and the capability of consecutive expansion; culture-expanded MSCs unavoidably consist of heterogeneous population of cells with differentially committed progenitors, whereas the degree of heterogeneity varies depending on the isolation technique, culturing protocols and media used, passage number as well as tissue origin [9–13]. In 2005, the International Society for Cellular Therapy (ISCT) issued a position statement for the nomenclature of mesenchymal stromal cells (MSCs) [14–16], clarifying that the term mesenchymal stem cell is not equivalent or interchangeable with MSC (mesenchymal stromal cell) as well as defining MSC when meeting minimal criteria; these include being plastic adherent; having trilineage differentiation potential (osteogenic, adipogenic, and chondrogenic); cell-surface expressing of CD90, CD105, and CD73 (positive, > 95%); and lacking cell surface antigens CD45, CD34, CD14 or CD11b, CD79α or CD19, and HLA-DR (negative, < 2%). Subsequently, the discovery that perivascular cells meeting the ISCT MSC minimal criteria led to a recent important paradigm shift in our understanding of in vivo identity of MSCs being perivascular pericytes [17, 18], which markedly diversifying the study and application of MSCs. Previously, investigational new cellular therapeutics were almost exclusively derived from BM [19]; however, in the past decade, approximately half of the new MSC products applied in clinical trials have been obtained from tissues other than BM, typically enriched with vascular structure [13].

**Fig. 1:**
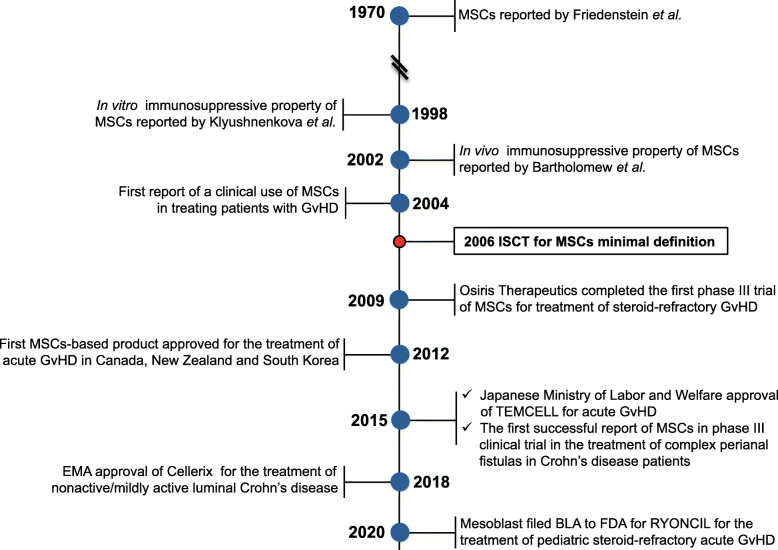
Timeline for major events in studies of the immunosuppressive effects of MSCs and the progress in clinical applications

Pioneering translational studies on the exploitation of the stem/progenitor properties of MSCs nonetheless revealed MSCs have the capacity to dampen inflammatory response, affecting the functionality of both adaptive and innate immune systems [11, 20–22]. MSCs produce extracellular vesicles (EVs), including exosomes and microvesicles, and a multitude of cytokines and growth factors capable of suppressing immune responses by inhibiting B and T cell proliferation, preventing monocyte differentiation and dendritic cells (DCs) maturation, meanwhile promoting generation of regulatory T cells, regulatory B cells, and M2 macrophages [23–25]. Such insight led to first clinical trials, which found transfusion of MSCs contributed to accelerating hematopoietic recovery following high-dose myeloablative chemotherapy and reversing steroid-resistant graft versus host disease (GvHD) [26], and actual current clinical value of MSCs is primarily derived from immunomodulatory properties (demonstrated in Fig. 1), [11, 27, 28]. Since the first clinical trial using MSCs as cellular pharmaceutical agents, numerous clinical trials have been conducted to test the efficacy of MSC-based therapy and over 10,000 of patients have been administered with allogeneic or autologous MSCs for the treatment of various diseases [21, 29] (Mesenchymal stem cells search at www.clinicaltrials.gov, accessed on 24 April 2020), including GvHD, myocardial infarction (MI), stroke, Crohn’s disease, multiple sclerosis (MS), amyotrophic lateral sclerosis (ALS), diabetes, lupus, arthritis, acute lung injury, Covid-19 [30], cirrhosis, and so on. Due to the accessibility, ease of isolation, and therapeutic efficacy, by far, the most prevalent source of human MSCs in clinical trials remains adult BM, followed by AT with an emergence of postpartum discarded tissues, such as umbilical cord, placenta, amniotic membranes, and cord blood [13]. Here we present an overview of the latest findings and medical translation of MSCs in preclinical studies and clinical applications.

## MSC therapy: biological properties supporting clinical use

Clinical trials exploring MSC therapy have been driven predominately by companies pursuing the development and commercialization of proprietary allogeneic MSC products, such as Mesoblast’s Remestemcel-L, Mesoblast’s Revascor, Athersys’ MultiStem, Stempeutic’s Stempeucel, Stemedica’s Stemdyne-MSC, Allocure’s AC607, Pluristem’s PLX-PAD and PLX-R18, TiGenix’s Darvadstrocel, and Orthofix’s Trinity Evolution. Briefly, pre-banked allogeneic MSCs derived from small groups of donors are subjected to culture expansion to generate therapeutic agents for treating allogeneic unrelated recipients [27, 31–34]. Though a uniform mechanism governing MSC-based therapy has not yet been demonstrated, the therapeutic potential of MSC deployment should be considered from bellow aspects: (i) paracrine effect by secreting soluble factors crucial for cell survival and proliferation, (ii) modulating immune responses, and (iii) migrating to the site of injury.

### Paracrine effects

MSCs do not persist following infusion [35–38]. The therapeutic benefits of MSC transplants are mostly attributable to the so-called hit-and-run mechanism mediated by the production of EVs and the secretion of cytokines, chemokines, and growth factors, to exert effects during the initial days following MSC injection. With the majority of cells dying within 48 h [39–42], long-term engraftment has been found very limited and ectopic tissue formation rarely reported. Using donor DNA/RNA analysis, bioluminescence tracking, and intravital imaging, engrafted MSCs were shown to be short-lived [38]. Recently, tissues from 18 patients who received MHC-mismatched or haplo-identical MSCs were systemically analyzed, and basically, no ectopic tissue was observed [36], although high levels of donor DNA (> 1/1000 cells) were found in one patient at multiple sites; however, this patient was unrepresentative given he was severely immunocompromised and septic and received MSC infusion 7 days before his death [36]. Since intravascular infusion is the most popular route for clinical MSC delivery, the mechanisms mediating the persistence of systemically infused MSCs has mostly been studied, revealing a large fraction of infused therapeutic cells being lost due to their triggering of instant blood-mediated inflammatory reaction (IBMIR) [43, 44], resulting in MSCs rapidly embolized and destroyed in the microvasculature [13, 44].

On the other hand, over the years, our understanding of MSC functionality has undergone another paradigm shift that MSCs yield therapeutic benefits largely via paracrine effects and stimulation of host cells rather than cell replacement [22]. It has becoming increasingly evident that the therapeutic actions of MSCs are broadly attributed to numerous biologically active soluble substances secreted by MSCs to deliver immunomodulatory, angiogenic, antiapoptotic, and antioxidative effects. For instance, MSCs secrete vascular endothelial growth factor (VEGF), fibroblast growth factor (FGF), hepatocyte growth factor (HGF), placental growth factor (PGF), monocyte chemotactic protein 1 (MCP-1), stromal cell-derived factor 1 (SDF-1), and angiopoietin-1 (Ang-1) that are critical for vascularization [45–52], enabling MSCs to ameliorate ischemia and chronic inflammation and facilitate wound repair [53–62]; MSCs synthesize and secrete B cell lymphoma 2 (BCL-2), survivin, VEGF, HGF, insulin-like growth factor-I (IGF-I), stanniocalcin-1(STC-1), transforming growth factor β (TGF-β), FGF, and granulocyte–macrophage colony-stimulating factor (GM-CSF), inhibiting cellular apoptosis and restoring tissue homeostasis [63–66]; or MSCs modulate immune response principally via suppressive mediators, such as prostaglandin E-2 (PGE-2), soluble human leukocyte antigen G5 (sHLA-G5), TGF-β, HGF, IL-10, IL-6, indoleamine 2,3-dioxygenase (IDO), nitric oxide (NO), inducible nitric-oxide synthase (iNOS), hemeoxygenase-1 (HO-1), galectin-1 (Gal-1), Gal-9, and TNFα stimulated gene 6 (TSG-6), [25, 29, 41, 67–73]; moreover, MSCs regulate migration via a variety of chemokines such as CCR1, CCR2, CCR4, CCR7, CXCR5, and CCR10 [74–77]. In all, MSCs mediated immunomodulation and regenerative activity is a redundant system and none of these molecules has an exclusive role. Depleting any one of these molecules would not result in a complete loss of the regulatory action it involved, and their relative contribution to the therapeutic effects of MSCs varies between different studies.

### Immunomodulation

The discovery that bone marrow-derived MSCs might suppress co-cultured immune cells led to the intensive investigations of the immunomodulatory properties of MSCs [20]. In 2002, Bartholomew et al. found MSCs capable of suppressing the proliferation of co-cultured leukocytes in a dose-dependent manner, and regardless of MSC-donor origin, such suppression could be induced and observed [78]. Moreover, the addition of IL-2 impaired MSC-mediated suppression, suggesting the process was partially reversible and MSCs did not induce T cell anergy [78, 79]. The immunosuppressive potential of MSCs was thereby tested in baboon skin allograft model in vivo, with MSCs intravenous injection immediately conducted after transplanting MHC-mismatched skin graft. The administration of donor-matched or third-party MSCs was shown able to extend the survival of the skin graft from 7 days (control without MSCs) to 11.3 and 11.8 days, respectively, indicating MSCs promote tolerance to transplanted tissues and the inhibition is not MHC restricted [80]. This new insight that MSCs have unique immunologic characteristics underlying their survival and growth in allogeneic or xenogeneic environments potentially opened the avenue for MSCs in the applications of treating various immunology related disorders [81].

**Fig. 2:**
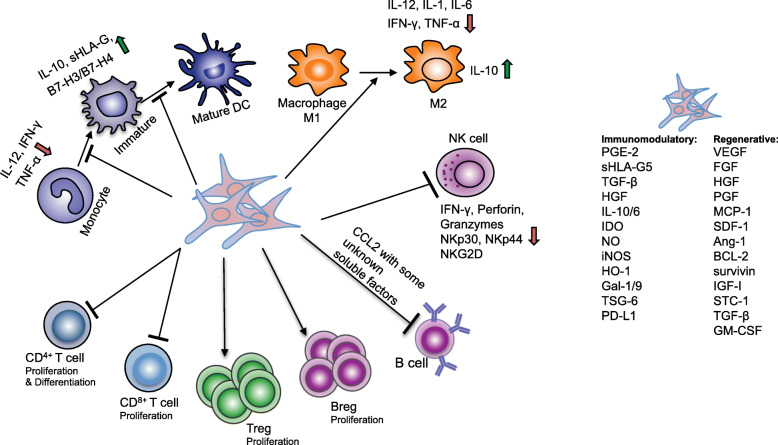
Mechanisms mediating immunomodulation. MSCs exert their effect on innate and adaptive immune systems via cell-to-cell interactions and immunomodulatory/regenerative factors. Depleting any one of these molecules would not induce a complete loss of its involved regulatory activities of MSCs and their relative contribution to the therapeutic effects varies between different studies. MSC-mediated immunomodulation and regenerative action is a redundant system, and none of these molecules has an exclusive role

MSCs exert immune tolerant phenotype by expressing very low levels of major histocompatibility complex (MHC) Class I surface antigens, accompanied by reduced expression levels of the major components of the antigen processing machinery (APM), and do not express MHC Class II antigens unless inflammatory signaling stimulated [27, 29], FasL or co-stimulatory molecules such as CD80 (B7-1), CD86 (B7-2), CD40, or CD40L [11, 80–83]. In addition, MSCs release sHLA-G, a non-classical MHC Class Ib antigen that is typically involved in the establishment of immune tolerance at the maternal-fetal interface, as well as express the co-inhibitory molecules B7-H1 (PD-L1) and B7-H4, to further prevent the immune attacking [84]. MSCs also dynamically express the Toll-like receptors (TLRs) 2, 3, 4, 7, and 9, affecting the pro- or anti-inflammatory properties of MSCs according to microenvironmental context. To fully explore MSC-mediated beneficial immunomodulation, particularly immunosuppression, in clinical application and implementation, the underlying mechanisms by which MSCs interact with the innate and adaptive immune cells shall be determined and are currently under in-depth investigation (summarized in Fig. 2).

#### Effect of MSCs on dendritic cells (DCs)

Dendritic cells maintain and modulate immune responses through dual effects. DCs can prime naïve CD4^+^ T cells polarizing towards Th1, Th2, or Th17 effector T cells and stimulate CD8^+^ T cytotoxic activities; meanwhile, DCs can induce the expansion of immunosuppressive CD4^+^ Treg cells and promote effector T cell apoptosis, enforcing immune tolerance [85–87]. Recent studies demonstrated that MSCs could block the differentiation and maturation of monocytes towards DCs and decrease the cell-surface expression of CD1-α, CD40, CD80, CD83, CD86, and MHC-II [70, 88, 89]. In the presence of MSCs, DCs also decrease the production and secretion of IL-12, interferon-γ (IFN-γ), and TNF while accelerating IL-10 release [88], resulting in impaired ability of antigen presentation. In addition, the immunosuppressive traits of DC, such as sHLA-G or B7-H3 and B7-H4, typically involved in the protection of allogeneic transplants, were also increased in the presence of MSCs [90]. Lately, Reis et al. report the exosomes/microvesicles of MSCs significantly enriched with immunomodulatory microRNAs, such as miR-21-5p, miR-142-3p, miR-223-3p, and miR-126-3p, were capable of distinctively undermining immature DCs antigen up-taking as well as halting DC maturation [91].

#### Effect of MSCs on natural killer (NK) cells

As sensors for microbial products and sentinels of virus infected cells, NK cells can kill non-self and transformed cells lacking MHC class I molecules, together with targeting cells expressing the ligands capable of activating NK cell surface receptors [92]. In contrast to cytotoxic T cells, NK cells do not require prior antigen exposure to mediate their cytolytic activity [92]. MSCs can be strong inhibitors of NK cells in terms of proliferation and cytotoxicity. It has been shown the presence of MSCs substantially reduced IL-2/15-induced NK cell proliferation, cytotoxicity, the production of IFN-γ, and the level of perforin and granzymes [93–95]. Furthermore, the expression of surface receptors, such as NKp30, NKp44, and natural-killer group 2 member D (NKG2D) typically involved in NK cell activation and target cell killing, were also downregulated. In addition, immunosuppressive secretors PGE-2, TGF-β, IDO, and sHLA-G constitutively produced by MSCs also contribute to MSC-mediated NK cell inhibition. However, the potent suppressive effects of MSCs were only apparent at high MSC-to-NK ratios [96, 97]. Both autologous and allogeneic MSCs have been found dissolved by cytokine-activated NK cells when sufficient activating receptors expressing on NK cells [98]. Incubation of MSCs with IFN-γ partially protected them from NK-cell-mediated cytotoxicity, suggesting a microenvironment rich in IFN-γ might favor MSCs inhibiting NK cells, whereas in the absence of IFN-γ, the balance would be tilted towards NK cells eliminating MSCs [92–96, 98–100]. Similarly, it has been shown that TLR4-primed MSCs were more resistant than unprimed MSCs to activated NK cell killing, and in contrast, no comparable protection was observed after TLR7/8-priming of MSCs [97, 101–103]. Taken together, the capability of MSCs exerting suppressive effects on NK cells or the susceptibility of MSCs to NK-cell-mediated cytotoxicity depends on the complex interaction between two types of cells and the ratios between them, as well as the microenvironmental context.

#### Effect of MSCs on macrophages

Macrophages are key players in initiating and controlling immune response with significant plasticity [104]. In the context of inflammatory environment, typically with high levels of TNF-α and IFN-γ, MSCs have been widely reported to promote macrophage polarization towards anti-inflammatory M2 phenotype, downregulating the secretion of pro-inflammatory cytokines while upregulating phagocytic activities and the release of tropic factors and IL-10; whereas in the absence of local inflammatory cues, MSCs induce the differentiation of macrophages towards pro-inflammatory M1 phenotype through secretion of IFN-γ and IL-1 as well as elevating the expression of CD40L on cell surface [105–107]. Some in vitro observations have been confirmed in in vivo studies, showing MSCs play an important role of educating macrophages to promote tissue repair during injury resolution. For instance, applying MSCs to treat renal damage after ischemia lessened the infiltration of macrophages and increased the proportion of M2 macrophages, combined with increased IL-1/6 production and secretion of IL-10 at ischemic sites [108]. In an animal model of skin wound healing, it has also been shown MSCs were capable of improving wound closure, dampening the M1 inflammatory response and promoting the induction of M2 macrophages [109].

#### Effect of MSCs on T lymphocytes

In adaptive system, MSCs can modulate the intensity of immune response by inhibiting antigen-specific T cell proliferation and cytotoxicity, as well as promoting the generation of regulatory T cell (Treg) [110]. It has been demonstrated that in vitro T lymphocyte proliferation induced by polyclonal mitogens, allogeneic cells, or specific antigen can be considerably inhibited by MSCs. MSCs are capable of promoting apoptosis of activated T cells via the Fas/Fas ligand pathway [111], as well as constitutively secreting inhibitory mediators such as B7-H4, HLA-G, PGE2, IDO, NO, and HO-1 [112, 113]. The release of these suppressive factors can be enhanced following stimulation of MSCs with TNF-α and IFN-γ [114–116]. Furthermore, several studies have reported the ability of MSCs to polarize T cells towards immunosuppressive regulatory phenotype, dampening inflammation [11, 35]. Particularly, soluble mediator IDO promotes the degradation of tryptophan to generate kynurenine and numerous other catabolites, which have been shown to contribute greatly to suppressing T cell proliferation and inducing Treg cells [117, 118]. Very importantly, fail-safe mechanism exists in MSCs’ actions, preventing excessive inhibition of T cell responses and host being vulnerable to infectious agents [11, 119]. MSCs can acquire distinct immunophenotypes through TLRs in accordance with microbe-associated molecular patterns in the microenvironment [11]. For instance, TLR4-primed MSC population exhibits a pro-inflammatory profile, and TLR3-primed MSC population delivers anti-inflammatory immunomodulation [120], enabling MSCs to influence the functionality of T cells depending on the microenvironment. It has been found MSCs may lose the ability to inhibit T cell proliferation following the exposure to pathogen-associated ligands and triggering the expression of TLR4 due to impaired Notch signaling [119, 121–123]. In short, pathogen-associated molecules could reverse the suppressive effects of MSCs on T cells and restore efficient T cell responses to pathogens.

#### Effect of MSCs on B lymphocytes

B cells are the second major cell population related to adaptive immune response, and it has been shown MSCs are capable of suppressing B cell proliferation, reducing plasmablast formation as well as promoting induction of regulatory B cells (Bregs) [124, 125]. Bregs have immunosuppressive properties usually mediated by IL-10 secretion, through which they provide immunological tolerance and convert effector CD4^+^ T cells into Foxp3^+^ Tregs [126]. In addition, MSCs have been shown to affect the chemotactic properties of B cells, as the CXCR4, CXCR5, and CCR7 in B cell expression were significantly downregulated in the presence of MSCs, along with declined chemotaxis to CXCL12/13, the CXCR4/5 ligands [125, 126]. Both cell-contact and secreted factors are needed for MSC modulation of B cells [127]. In particular, metalloproteinase processed CC-chemokine ligand 2 (CCL2) released by MSCs was found to suppress the activity of signal transducer and activator of transcription 3 (STAT3) signaling pathway and thus inhibit immunoglobulin synthesis in B cells via compromising PAX5 function [128]. Several other signaling pathways, such as B lymphocyte-induced maturation protein 1 (Blimp1), p38, extracellular response kinase 1/2 (ERK1/2), and PI3K/AKT/mTOR signaling, also negatively modulate B cell activation [129]. However, similar to mediating T cell activity, inadequate inflammatory signal-activated MSCs may support proliferation and differentiation of antibody-releasing B cells [130, 131], meaning MSC suppressive effects relying on the strength of the inflammatory stimulation to yield the plasticity of MSC immunomodulation [10, 132, 133].

### Homing and hemocompatibility consideration

One of the key benefits of MSC-based therapies is their ability to preferentially migrate to damaged tissues exhibiting inflammation [134]. Although in situ administration can directly achieve this goal, this possibility can be hampered by the anatomical location of the damaged tissue or by the systemic nature of the illness [11]. Basically, in non-systemic homing, MSCs are transplanted locally at the target tissue and then guided to the site of injury via a chemokine gradient, whereas in systemic homing, systemically administered MSCs must undergo a multistep process to exit circulation and migrate to the injury site, supporting functional recovery [74].

**Fig. 3:**
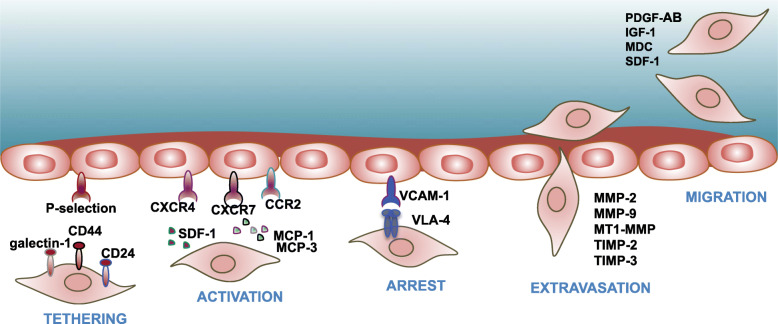
Overview of the molecular mechanisms facilitating each step of MSCs homing

The initial tethering step (illustrated in Fig. 3) is mediated by selectins expressed by endothelial cells and CD44 expressed by MSCs [135–137]. The interaction between these two factors facilitates MSCs to begin rolling along the vasculature wall. However, as most understanding of migration and homing mechanisms derived from studies evaluating leucocytes migrating into inflamed tissues, the fact that MSCs do not express the hematopoietic cell E-/L-selectin ligand (HCELL) [135], a specialized glycoform of CD44 on the migrating cell, as well as the P-selectin glycoprotein ligand-1 (PSGL-1) [135], raises the question of which selectin exactly employed by MSCs. One study has identified galectin-1 [138] as one such candidate, and another study has identified CD24 as a potential P-selectin ligand [139]. Following the initiation of tethering step, activation step is facilitated typically by G protein-coupled chemokine receptors in response to inflammatory signals. It has been extensively demonstrated that the CXCR4-SDF-1 axis is critical for this step [140, 141]. However, the expression of the chemokine receptor CXCR4 on MSCs is inconsistently observed, suggesting that other receptors are also involved, for some groups did not observe expression of the receptor while other studies demonstrated overexpression of CXCR4 on MSCs affected migration in response to SDF-1 [142–144]. Indeed, aside from CXCR4, MSCs express CCR1, CCR4, CCR7, CCR10, CCR9, CXCR5, and CXCR6 [75, 76], of which their roles remain to be elucidated in detail. Integrins play a critical role in the activation-dependent arrest in the third step of homing, with integrin α4 and β1 subunit combined to form very late antigen 4 (VLA-4) that capable of interacting with vascular cell adhesion molecule 1 (VCAM-1) to enable arrest [145]. Inhibiting integrin β1 subunit has been shown to abolish MSC homing [141, 145, 146]. In the final step, MSCs must travel through the basement membrane underlying endothelial cells, which requiring various matrix metalloproteinases (MMPs) to cleave the components of the connective tissue [145]. As the gelatinases MMP-2 and MMP-9 preferentially degrade collagen and gelatin, they play significant roles in this step [147–149]. It has also been shown this step is regulated by tissue inhibitor metalloproteinase 3 (TIMP-3), TIMP-2, and membrane type 1 MMP (MT1-MMP), and very likely, there are more molecular interactions involved in MSC extravasation [150, 151]. Finally, guided by chemotactic signals, MSCs must migrate to the site of injury. MSCs migrate towards various signals, including the growth factors platelet-derived growth factor-AB (PDGF-AB) and insulin-like growth factor 1 (IGF-1), and to a lesser extent, the chemokines MDC and SDF-1 [74].

As mentioned above, the majority of infused therapeutic MSCs are lost due to their triggering of IBMIR, mediated primarily by the innate coagulation and complement cascade system, thus assessing and controlling hemocompatibility should be considered and implemented for safer and more effective MSC therapies. Firstly, eliciting IBMIR by MSC products is correlated with their expression of tissue factor (TF/CD142) [43, 152, 153], which has been found to be a potent trigger of coagulation. The pro-coagulant effect of MSCs increases as TF expression elevated, and MSC’s TF expression has been found upregulated as cell passage increases [44]. Secondly, the complement cascade capable of initiating innate immune attack and MSC embolization is another major leading cause of IBMIR [43, 153, 154]. Therefore, Moll et al. suggested hemocompatibility profiling and testing of complement compatibility should be conducted in vitro as well as in vivo, in addition to standardization of cell reconstitution, supplementing the formulation with additives such as anticoagulants, to optimize the stability, tolerability, and performance of MSC products [13].

## MSC-based cell therapy

MSCs represent a significant fraction of the current efforts to develop cell-based treatments for a range of diseases, and the number of clinical trials has been substantially rising since the last decade. Querying the ClincalTrials.gov international database provides important insight into the development of MSC applications in clinical trials. Currently, there are 1094 registered clinical trials in different clinical phases throughout the world (Accessed 24 April 2020). Among 1094 registered trials, 320 trials are completed and 77 studies are in the status of being withdrawn/terminated/suspended, whereas the rest are in the active status; 208 studies are recruiting and 104 studies not yet starting to recruit; most of these trials are phase I–II studies (847 studies), and less than 6% studies in the phase III or the combination of phase II/III (64 studies), whereas very small numbers of these trials are in phase III or phase III/IV (6 studies). To date, 149 trials are registered for immune-related disease, including 47 for GvHD, 25 for Crohn’s disease, 26 for type 1 diabetes, 29 for MS, and 14 for lupus, as well as 146 trials for cardiovascular diseases, 54 for liver disorders, 82 for respiratory disorders, 22 for Covid-19, 33 for spinal cord injury, 18 for kidney failure, 62 for skin diseases, 13 for Alzheimer disease (AD), 7 for Parkinson disease (PD), and rest are for other conditions. Here, we discuss some of the recent clinical developments, focusing on the registration trials paving the path for the commercialization of MSCs for GvHD and Crohn’s disease.

### MSCs in GvHD

Graft-versus-host disease (GvHD) accompanies allogeneic hematopoietic stem cell transplantation (HSCT) in many patients and is associated with high morbidity and mortality [155]. GvHD is a form of rejection characterized by the attack of transplanted cells to host tissues and organs. It has been estimated that nearly 50% of all allogeneic blood and marrow transplant patients develop acute GvHD [156]. Liver or gastrointestinal involvements occur in up to 40% of all patients with acute GvHD and are associated with the greatest risk of death, with mortality rates of up to 85%. Currently, corticosteroids remain to be the gold standard for the initial treatment of acute GvHD with the response rate of 50–80% [155, 157, 158]. However, it has been only 10–30% chance of long-term survival for the patients whose initial therapy failed. Immunoregulation capability of MSCs suggests their potential application in lessening GvHD severity and facilitating the engraftment of HSCs [26]. In 2004, Le Blanc et al. first transplanted haploidentical MSCs in a 9-year-old boy with severe treatment-resistant grade IV acute GvHD of the gut and liver [26]. One-year follow-up observations reported a remarkable clinical response and improvement.

Subsequently, the first major industry-sponsored phase III trial of allogeneic, BM MSCs for the treatment of steroid-refractory GvHD (NCT00366145) was conducted and completed by Osiris Therapeutics. The MSCs were sourced from healthy volunteers and then manufactured and cryobanked as allogeneic MSC product of Prochymal™. For the primary endpoint, the overall response rate was 82% with Prochymal versus 73% for placebo, failing to demonstrate a significant advantage of Prochymal over placebo. In retrospective, subset analysis revealed the improvement in children, which ultimately led to its approval in Canada via a Notice of Compliance with Conditions (NOC/c). In 2013, the Prochymal assets were acquired by Mesoblast that sponsored adaptive clinical trial of MSCs in pediatric GvHD (NCT02336230), and the cellular product was renamed as Remestemcel-L. Based on various observations obtained on previous clinical trial NCT00366145, children responded better than adults to allogeneic MSCs overall, after onset treating patients early on was better than delayed intervention, gut and liver GvHD were more responsive than skin type, multiple modifications were implemented in NCT02336230, including age of inclusion, severity of disease, starting time for MSC transfusion, the definition of response, and as exclusion of skin-only GvHD, while maintaining applying identical MSC products and dosing schemes in both clinical trials. The recruitment was completed in December 2017, and in February 2018, it was announced treatment with Remestemcel-L significantly improved the overall response rate at day 28 (69%) compared with the protocol-defined historical control rate of 45% (*p* = .0003). In addition, an overall survival rate at 6 months for the MSC-treated group was shown to be 69%, a considerable improvement compared with the historical survival rates of 10–30% in patients with steroid refractory grade III and IV GvHD. With these results [159–163], Biologics License Application (BLA) for Remestemcel-L (Ryoncil™) for the treatment of children with steroid-refractory acute GvHD has been lately accepted for the priority review by the US FDA, which has set a Prescription Drug User Fee Act (PDUFA) action date of September 30, 2020, and if approved, Remestemcel-L will be commercially available in the USA.

### MSCs in Crohn’s disease

Crohn’s disease is a chronic inflammatory disorder of gastrointestinal tract [164, 165]. In the early 1990s, Crohn’s disease patients were reported to experience relief from their inflammatory bowel disease following infusion of HSCs, and subsequently, HSC transplantation has been developed for refractory patients that do not respond to conventional treatments involving steroids or immunosuppressive agents or anti-TNF therapy [166–168]. However, serious adverse effects accompanied HSC transplantation treatment [169–172], which triggered the attempts to employ allogeneic or autologous MSCs for Crohn’s disease therapy. Early phase studies reported encouraging observations and autologous BM-MSC exhibited efficacy with improved Crohn’s disease activity index scores [173–176]. More recently, Cellerix sponsored the clinical trial NCT00475410 of adipose-derived autologous MSCs for the treatment of complex perianal fistulas in patients without inflammatory bowel disease. The study completed enrollment of 214 subjects in 2009; however, examining the primary endpoint of sustained closure and healing of fistulas 6 months after treatment failed to demonstrate that the adipose MSC application was superior to applying fibrin glue alone. In 2011, Cellerix was acquired by TiGenix which next sponsored an adaptive phase III trial with various changes: replacing autologous with allogeneic adipose MSCs, increasing the cell dose considerably, no fibrin glue matrix was applied, and only enrolling the patients with Crohn’s disease.

In 2015, this TiGenix-sponsored randomized, double-blind, placebo-controlled phase III clinical trial (NCT01541579) [177, 178] was completed, reporting statistically significant improvement over control in the treatment of complex perianal fistulas in Crohn’s disease patients, which represents the first unambiguously successful use of MSCs in an advanced clinical trial [27]. Significant difference was observed in patients treated with allogeneic adipose MSCs (50%) versus control (34%) after 24 weeks. Moreover, less treatment-related adverse events were observed in the allogeneic adipose MSCs group, with the therapeutic benefit and the good safety profile maintained after 1 year of treatment. In March 2018, TiGenix received the approval of its first allogeneic adipose MSC product Alofisel® (formerly Cx601/darvadstrocel) by the European Medicines Agency (EMA) to treat complex perianal fistulas in adult patients with nonactive/mildly active luminal Crohn’s disease.

### MSCs in cardiovascular diseases

Cardiovascular diseases (CVDs) affecting both heart tissue and circulatory system, especially blood vessels, are today leading cause of mortality worldwide [179–182]. As the least regenerative organ in the human body, scar forms following myocardial infarction (MI) due to the loss of cardiomyocytes and being replaced with fibroblasts, leading to contractile dysfunction of the heart [183, 184]. The potential capability of MSCs differentiating into mesoderm- and non-mesoderm-derived tissues, their immunomodulatory and regenerative effects, and overall availability have rendered MSCs one of the most intensively investigated and clinically tested cell type for CVDs. Autologous and allogeneic MSCs were employed to treat numerous acute as well as chronic cardiomyopathies in preclinical trials [185–189], suggesting safety, effectiveness of reduction in arrhythmias, improvement in functional status and increased ejection fraction. For clinical trials, MSCs have been used to treat pediatric cardiomyopathy, congenital heart diseases (hypoplastic left heart syndrome), refractory angina, myocardial infarction, and chronic ischemic cardiomyopathy [190–194]. Comparing the results of preclinical studies to those of clinical trials, only marginally beneficial effects could be observed in the latter [183, 184, 195–197]. In comparison, chronic ischemic cardiomyopathy could be more easily yielding consensus with preclinical observations thanks to various perceptible beneficial effects, including anti-fibrotic action, neo-angiogenesis, and contractility enhancement [198–204].

Autologous MSC-based therapy has emerged as a fast developing treatment modality for CVDs. In attempt to evaluate the safety and effectiveness of autologous MSCs, a total of 31 patients suffering from stable coronary artery disease and refractory angina received the intramyocardial injection of autologous marrow-derived MSCs. Significant improvement in left ventricular function and exercise capacity was reported in this study, in addition to various improvements in clinical symptoms [205]. Subsequently, these positive results were confirmed by a placebo controlled phase II trial evaluating the treatment of patients with chronic ischemic heart failure via the intramyocardial delivery of autologous MSCs. A total of 60 patients were randomized to receive the injection of either MSCs or placebo, and after 12 months, MSC infusion appeared to have induced the regeneration of damaged myocardial tissue along with the improved functional capacity of heart [204]. In a recently completed sham-controlled phase III trial for the treatment of chronic advanced ischemic heart failure (NCT01768702) [206], autologous marrow-derived MSCs were polarized to undergo lineage specification to acquire cardiopoietic phenotype and then administrated via endomyocardial injections. The primary endpoint results were unable to demonstrate significant difference between MSC intervention group and placebo after 39 weeks. However, the analysis of ventricular remodeling 52 weeks after treatment revealed that reverse remodeling was evident in patients receiving cardiopoietic MSCs, suggesting some beneficial effects [27, 204, 207].

### MSCs in autoimmune diseases

MSCs are also employed to alleviate various debilitating autoimmune disorders, such as systemic lupus erythematosus (SLE), type 1 diabetes, and MS, among others [29, 32, 129, 208]. SLE is a chronic autoimmune multiorgan-involved inflammatory disorder, characterized by aberrant activation of effector T lymphocytes and development of autoantibodies to nuclear antigens [209–213]. Lupus nephritis being the most prevailing organ manifestation is the major cause of mortality and morbidity in SLE patients [211, 214, 215]. For the past 20 years, no novel prospective clinical trial has demonstrated its effectiveness for SLE and there is an urgent unmet need to develop more effective therapies based on immunomodulatory and immunosuppressive strategies with fewer side effects, for which MSC application in the treatment of SLE has been explored. Recently allogeneic bone marrow or umbilical cord-derived MSCs were applied to the treatment of 87 refractory SLE patients [216] in open-label single armed phase I/II clinical trial (NCT01741857). Their results showed 32.5% of patients reached a significant clinical efficacy with a well-tolerated safety and a dramatic decline in disease activity scores review [129]. Of note, the promising report derived from NCT01741857 has not yet led to any advanced phase of clinical trial. Currently, there are four active clinical trials with a targeted enrollment of 137 patients listed in the ClinicalTrial Database.

Type 1 diabetes is a form of diabetes mellitus that results from T cell-mediated autoimmune destruction of insulin-producing pancreatic β cells as well as decreasing insulin secretion owing to anti-islet autoantibodies [217, 218]. Insulin application and blood glucose control is the standard therapeutic methods for type 1 diabetes. However, adverse effects are accompanied by intense insulin injection, and maintaining normal glycemic levels is often difficult and associated with increased frequency of hypoglycemic episodes, prompting new strategies to tackle this emerging global epidemic [219, 220]. It has been demonstrated that MSCs could differentiate into glucose competent pancreatic insulin-producing cells (IPCs) in vitro as well as in vivo [221–223], in addition to the capacity to regulate the immunomodulatory effects. It has been showed that transplantation of BM cells and MSCs in sublethally irradiated diabetic mice could regenerate islet β cells and reinforce glycemic control of type 1 diabetes; in their study, combined BMCs and MSCs infusion appeared to be synergistic [224, 225]. Recently, in a randomized controlled open-label phase I/II clinical trial (NCT01374854), cotransplantation of allogeneic Wharton’s jelly UC-MSC combined with autologous bone marrow mononuclear cell was shown to improve insulin secretion and reduce insulin requirement compared with baseline and the standard treatment controlled group [224]. Currently, there are six active clinical trials with a targeted enrollment of 172 patients listed in the ClinicalTrial Database.

MS is a chronic autoimmune condition due to the demyelination of the central nervous system (CNS). MS is one of the most prevalent autoimmune diseases mediated by pathogenic CD4^+^ T cells in the CNS. Although there are 2.3 million people influenced by this disease [226, 227], there is no effective means to stop the progression of disease and induce remyelination. Immunomodulation featured treatments have been exploited to ameliorate inflammation and neurodegeneration, with some MSC transplantation studies showing improved outcome in the MS animal model of experimental allergic encephalitis (EAE) [228, 229]. In addition, several disease models demonstrate axonal neuroprotection following MSC application, presumably achieved by MSC-mediated immunosuppression and the production of regenerative neurotrophic/growth factors [230–234]. Currently, there have been 29 clinical trials of MS registered on the ClinicalTrial Database utilizing MSC therapy as medical interventions, and based on completed phase I/II trials, autologous BM MSCs are suggested to be neuroprotective and safe in treating MS patients [235].

## Future perspective

As Pittenger et al. lately described that MSCs were initially started as “a riddle wrapped in a mystery, inside an enigma” [29], years of research have shown MSCs are capable of exerting profound immunomodulatory and regenerative action, making MSC-based treatment one of the most promising and intensely pursued cellular therapies. At present, more than 1000 MSC clinical trials have been registered globally, enabling meta-analysis to demonstrate MSC application safety [22]. However, the clinical efficacy and our understanding of the underlying molecular mechanism for a variety of pathological conditions remain to be improved. The combination of rational selection of subjects according to clinical and biological parameters and better understanding of cell manufacturing, banking, and point of care deployment is key to yield an optimal short- and long-term therapeutic benefit [27]. Furthermore, preclinical data support the notion that the use of priming MSCs either by use of pharmaceutical agents or cytokines, genetic engineering, or reprogramming MSCs prior to infusion enhances their pharmaceutical potency and hemocompatibility, encouraging novel translational strategies. Moreover, there are currently more than 200 preclinical investigations and a few clinic studies being carried out to exploit the immunosuppressive and immunomodulatory properties of EVs derived from the MSCs [236]. In comparison to whole cell-based therapies, MSC-EV-related therapeutics promotes concept of cell-free “cell therapy 2.0” [237], given EV exhibiting specific advantages for patient safety such as lower propensity to trigger innate and adaptive immune responses and inability to directly form tumors. Nonetheless, various important questions regarding EV standardization, MoA underlining EV transmitted biological information in the form of proteins, glycoproteins, lipids and ribonucleic acids, and cost-effective production must be methodically addressed [236–240].

In conclusion, the pace of the clinical trials based on MSC-mediated therapies is outstripping the progress in its basic research, presenting a great challenge of establishing guidance but also creating an opportunity to deepen our understanding of therapeutic MSCs. To achieve optimal stability, tolerability, and performance of next-generation MSCs therapies, efforts are underway to improve product design and delivery, safety and potency assessment pre- and post-treatment, and the understanding of the exact MoA with the advancement of transcriptomic, proteomic-profiling technology.

## Data Availability

The data sets supporting the results of this article are included within the article.

## Abbreviations

MSCs: Mesenchymal stem cells
BM: Bone marrow
ISCT: International Society for Cellular Therapy
HLA-DR: Human leukocyte antigen-DR isotype
EV: Extracellular vesicle
DCs: Dendritic cells
GvHD: Graft versus host disease
MI: Myocardial infarction
MS: Multiple sclerosis
ALS: Amyotrophic lateral sclerosis
hMSCs: Human mesenchymal stem cells
AT: Adipose tissue
PT: Perinatal tissue
IBMIR: Instant blood-mediated inflammatory reaction
VEGF: Vascular endothelial growth factor
FGF: Fibroblast growth factor
HGF: Hepatocyte growth factor
PGF: Placental growth factor
MCP-1: Monocyte chemotactic protein 1
SDF-1: Stromal cell-derived factor 1
Ang-1: Angiopoietin-1
BCL-2: B cell lymphoma 2
IGF-I: Insulin-like growth factor-I
STC-1: Stanniocalcin-1
TGF-β: Transforming growth factor β
GM-CSF: Granulocyte–macrophage colony-stimulating factor
PGE-2: Prostaglandin E-2
sHLA-G5: Soluble human leukocyte antigen G5
IL: Interleukin
IDO: Indoleamine 2,3-dioxygenase
NO: Nitric oxide
iNOS: Inducible nitric-oxide synthase
HO-1: Hemeoxygenase-1
Gal-1: Galectin-1
TNF: Tumor necrosis factor
TSG-6: TNFα stimulated gene 6
CCR: C-C motif chemokine receptor
CXCR5: C-X-C motif chemokine receptor type 5
MHC: Major histocompatibility complex
APM: Antigen processing machinery
TLRs: Toll-like receptors
IFN-γ: Interferon-γ
NK: Natural killer
NKG2D: Natural-killer group 2 member D
Treg: Regulatory T cell
Breg: Regulatory B cells
CCL2: CC-chemokine ligand 2
STAT3: Signal transducer and activator of transcription 3
PAX5: Paired box 5
Blimp1: B lymphocyte-induced maturation protein 1
ERK1/2: Extracellular response kinase 1/2
HCELL: Hematopoietic cell E-/L-selectin ligand
PSGL-1: P-selectin glycoprotein ligand-1
VLA-4: Very late antigen 4
VCAM-1: Vascular cell adhesion molecule 1
MMP: Matrix metalloproteinase
TIMP-3: Tissue inhibitor metalloproteinase 3
MT1-MMP: Membrane type 1 MMP
PDGF-AB: Platelet-derived growth factor-AB
IGF-1: Insulin-like growth factor 1
MDC: Macrophage-derived chemokine
AD: Alzheimer disease
PD: Parkinson disease
HSCT: Hematopoietic stem cell transplantation
NOC/c: Notice of Compliance with Conditions
BLA: Biologics License Application
PDUFA: Prescription Drug User Fee Act
EMA: European Medicines Agency
CVDs: Cardiovascular diseases
SLE: Systemic lupus erythematosus
IPCs: Insulin-producing cells
CNS: Central nervous system
EAE: Experimental allergic encephalitis
